# Possible Role of –374T/A Polymorphism of RAGE Gene in Longevity

**DOI:** 10.3390/ijms141123203

**Published:** 2013-11-21

**Authors:** Colomba Falcone, Sara Bozzini, Anna Colonna, Benedetta Matrone, Edoardo Maria Paganini, Rossana Falcone, Gabriele Pelissero

**Affiliations:** 1 Interdepartimental Center for Research in Molecular Medicine (CIRMC), University of Pavia, Via Taramelli 24, Pavia 27100, Italy; E-Mails: sara-84@tiscali.it (S.B.); roxvieste@hotmail.it (R.F.); 2 Department of Cardiology, Istituiti Clinici di Pavia e Vigevano University Hospital, Via Parco Vecchio 27, Pavia 27100, Italy; E-Mails: anna.mcolonna@gmail.com (A.C.); betty.matrone@gmail.com (B.M.); johnny2@hotmail.it (E.M.B.); g.pelissero@grupposandonato.it (G.P.); 3 IRCCS San Donato Hospital, San Donato Milanese 20097, Italy

**Keywords:** receptor for advanced glycation end products, 374T/A polymorphisms, longevity

## Abstract

Demographic and social changes in the last decades have resulted in improvements in health and longevity. The survival of elderly people has improved significantly and thus centenarians are becoming the fastest growing population group. Environmental, genetic, and accidental factors have influenced the human life span. Researchers have gained substantial evidence that advanced glycation end products may play an important role in the processes of physiological aging. The aim of the present study was to investigate any differences in the frequencies of –374T/A polymorphism in subjects aged >90 years and in middle-aged individuals. We observed association between the A allele and genotype homozygous for this allele (AA) with a longer life expectancy in the male population. In particular, there was a prevalence of AA genotype and A allele in long-living subjects and a prevalence of the allele T in middle-aged subjects, indicating a possible protective role of the allele A to aging. In conclusion, our results support the hypothesis that longevity is the result of a good functioning of the immune system and a presumable hyper-expression of variants of anti-inflammatory genes of immunity. The differences in the genetic regulation of inflammatory processes may influence the presence of age-related disorders.

## 1. Introduction

During the last decades, demographic and social changes have resulted in improvements in health and longevity. The survival of elderly people has improved significantly and thus centenarians are becoming the fastest growing population group [1]. Environmental, genetic, and accidental factors influenced the human life span [2]. The numerous researches on genes involved in oxidative stress, lipid and glucose metabolism, inflammation, DNA damage and repair, axis of growth hormone and insulin-like growth factor [3] are still conflicting; it may be suggested that the combination of numerous fixed genetic variants will be necessary for someone to achieve exceptional longevity [4]. International research has focused its attention on centenarians as excellent study models for biological mechanisms of aging [5].

Researchers have gained substantial belief that advanced glycation end products (AGEs) may play an important role in the processes of physiological aging [6]. AGEs could be involved either through the formation of free radicals, or through molecular interaction with their specific surface receptor (RAGE) [7]. The recruitment of the receptor RAGE by AGE leads to an increase in oxidative stress inducing also a state of endothelial cell activation [8]. Upon binding with its ligands, RAGE quickly becomes over-expressed and becomes part of a positive-loop feedback mechanism that results in a destabilizing effect on homeostasis phone [9].

The –374T/A RAGE promoter polymorphism leads to reduced binding of a nuclear factor to a regulatory element of the RAGE gene promoter. Transcription factor binding assays revealed that the introduction of the –374 A allele cause the abolition of a nuclear protein binding site, supporting the role of these polymorphisms in affecting RAGE transcriptional repression [10].

The aim of the present study was to investigate any differences in the frequencies of –374T/A polymorphism in subjects aged ≥90 years and in middle-aged individuals.

## 2. Results

The characteristics of subjects included in the study were listed in Table 1. The population in this study consists of 254 Caucasian subjects (170 women and 84 men): subjects aged ≥90 years were included in the Long-living Group (*n* = 119) and subject aged 18 to <80 years were used as Control Group (*n* = 135). The age of long-living subjects group ranged between 90 and 106 (mean ± standard deviation: 93 ± 3). The mean age of the control group was 62 ± 10.

Levels of total cholesterol and glucose were significantly higher in subject aged ≥90 years (*p* = 0.0029 and *p* = 0.01 respectively). HDL cholesterol and triglycerides levels were not significantly different between cases and controls. As regards the cardiovascular risk factor, we found no differences in hypertension frequencies but we found that subjects having ever smoked was significantly lower in people more than 90 years old compared to controls (4% *versus* 19%, *p* < 0.01).

The genotypic and allelic distributions of the –374 T/A polymorphism are reported in Table 2. The distributions of the frequencies of each genotype are in agreement with those predicted by the Hardy-Weinberg equilibrium. In our study, we did not find significant differences between middle-aged individuals and the long-living group.

We also performed stepwise regression analysis to determine whether this association was independent from the effect of common cardiovascular risk factors and current treatment. The results showed that RAGE polymorphism remained related to the manifestation of heart disease, even after adjusting for these confounding variables. The correlation analysis, performed to evaluate which clinical-instrumental parameters were correlated to RAGE –374T/A polymorphism, showed that gender significantly correlated with polymorphism.

The number of women is larger among long-living subjects than in the middle-aged group. When categorized by sex, no differences were found about allelic and genotypic frequencies in female subjects. However, the frequency of homozygous AA genotype was higher in male subjects aged ≥90 years than in subjects aged <80 years. In particular, AA genotype was present in 18% of middle-aged individuals and in 43% of long-living subjects (*p* = 0.028). There was a significant reduction in frequency of heterozygous AT genotype in subjects aged ≥90 years compared to subjects aged <90 (*p* = 0.023). No differences were found as regards the TT genotype. Concerning allelic frequencies, we observed a greater frequency of A allele in the long-living group and a greater frequencies of T allele in middle-aged individuals, although it did not reach a statistical significance (Table 3).

## 3. Discussion

Demographic and social changes in the past decades have resulted in improvement both in health and in longevity. Centenarians represent excellent models to research biological mechanisms of aging and to study the genetic background. Among the various theories of aging proposed until today, the hypothesis that it is a process dependent on genetic factors is gaining increasing credit. This is based essentially on the fact that the duration of life of children born of long-living individuals is higher than that of children born of not long-living parents [11] and that the co-existence of longevity in identical twins is about two times higher than in fraternal twins [12].

Numerous genes would appear to contribute to the determination of longevity and aging; some would appear to increase susceptibility to age-related diseases and early death, while other genes would appear to have a role in slowing the aging process and thus contributing to longer life [13].

The present study investigated the presence of possible correlation between the –374 T/A polymorphism in the promoter region of the RAGE gene and longevity. We did not find any association between RAGE –374T/A allele and genotype with age in the entire population. However, in the male population, we observed an association between the A allele and genotype homozygous for this allele (AA) with a longer life expectancy. In particular, there was a prevalence of AA genotype and A allele in long-living subjects and a prevalence of the allele T in subjects with middle-age, indicating a possible protective role of the allele A to aging.

Some new studies showing that the aging process in both genders is gender-specific. Recently, Makrantonaki E, *et al.*, documented the gender-independent differences in the expression profiling of aged skin between young and elderly European Caucasian adults when circulating hormones reach their maximum levels in blood and after the onset of hormone decline, accordingly [14]. Previously, Ali SS, *et al*. explored the association between oxidative stress and gender-specific aging in C57BL6 mice, in which females are the shorter-lived gender. They demonstrated that differences in ROS homeostasis contribute to gender divergence in survival, but also suggest that mitochondrial superoxide production may not be primarily responsible for gender differences in lifespan [15].

Hofmann B, *et al.* demonstrated that skin autofluorescence and pulse wave velocity as non-invasive parameters significantly correlate with the AGE contained in graft material and therefore are strong predictors of vessel AGE modifications in patients with coronary heart disease. Whether the analysis of the skin autofluorescence leads to an improvement of the risk stratification in patients suffering from cardiovascular disease has to be further tested [16].

In our previous study, we found that the AA genotype seemed to correlate with the age of onset of myocardial infarction: patients with this genotype showed a 5 years higher mean age of onset than patients with at least one T allele, indicating that homozygosis for the A allele could play a protective role in the pathogenesis of the disease [17]. Our research group has also shown the beneficial influence of the homozygous genotype AA with respect to the development and progression/restenosis after percutaneous myocardial revascularization (PTCA) of advanced atherosclerotic lesions [18–20].

Women live longer than men and make up a large proportion of the older population. Whether genetic factors play more or less of a role in men or in women is a matter open to debate. Estrogens and phyto-estrogens up-regulate expression of antioxidant enzymes through estrogen receptor and MAPK activation, which activate the NF-κB (nuclear factor kappa-light-chain-enhancer of activated B cells) signaling pathway, resulting in the up-regulation of expression of longevity-related genes [21]. The association of A allele and longevity, found in the present study, seem to confirm the role of AGE-RAGE interaction in human age-related endothelial dysfunction.

The current study is a candidate-gene association study that compared long-living subjects with middle-aged subjects with no possible prediction of their future longevity. The mechanisms underlying the association between the studied polymorphism and longevity cannot be directly inferred from the present study but it can be assumed that the reduction of transcription of RAGE gene associated with AA genotype generate a decreased expression of the receptor on the cell surface and consequently a decrease in the pro-inflammatory signal in male subjects. The correct trigger axis RAGE-ligand results in a cell phenotype in which the activation of transcriptional factor NF-κB leads to the overproduction of pro-inflammatory mediators [22]. Cytokines, for example, have a key role in maintaining homeostasis of lymphocytes, in fact, alterations in the sequence of several genes coding for cytokines are associated with longevity [23–25]. A recent study by Balistreri *et al.* [26] found that male centenarians are characterized by the presence of allelic variants that suppress inflammation, so they seem better equipped in the defense of the major age-related disorders.

The aging process is a universal, intrinsic, progressive accumulation of deleterious changes in cells and tissues that increases morbidity and leads to death. The immune system seems to be involved in the chronic oxidative and inflammatory stress conditions of aging, according to the recent theory of oxidation-inflammation to explain the aging process [27]. Thus, human ageing can be explained at least in part by the concept of “inflamm-ageing” according to which ageing can be driven by the pro-inflammatory cytokines and other inflammatory mediators produced by the innate immune system [28]. In longevity, a higher frequency of genetic markers associated with a reduced proinflammatory ability seems to counteract the onset of the main age-related inflammatory disorders. It was demonstrated that Centenarians are enriched in “good” genotypes and show opposite frequencies of “bad” genotypes in comparison with patients affected by major age-related pathologies [29].

## 4. Methods

### 4.1. Study Population

The population in this study consists of 254 Caucasian subjects (170 women and 84 men). Subject aged 18 to <80 years, no personal history of coronary artery disease, no diabetes mellitus, no high alcohol consumption, professional athleticism and any chronic disease were used as control group (middle-aged group, *n* = 135). Subject aged ≥90 years who had MMSE (Mini Mental State Examination) score in the top 75th percentile [30] were included in the long-living group (*n* = 119). This MMSE cut-off has previously been used to characterize individuals as having “intact cognition” [31]. Patients with chronic inflammatory or autoimmune diseases and with known malignancies and renal or hepatic impairment were excluded from the study.

Patients included in the study underwent the following investigations: an accurate anamnesis with identification of the major cardiovascular risk factors; cardiology consultation including check of baseline blood pressure; peripheral venous blood sample for the investigation of the presences of RAGE –374T/A polymorphism. The cardiovascular risk factors were defined as follows: hypertension (systolic pressure > 140 mmHg or diastolic pressure >90 mmHg or antihypertensive therapy), tobacco smoke, hyperlipidemia, and diabetes mellitus. With regard to cigarette smoking, subjects were grouped under the headings “always” or “never”: in the former group were included subjects who had smoked daily for at least 1 year. We considered dyslipidemic patients with cholesterol levels above 200 mg/dL or in treatment with lipid-lowering drugs. The diagnosis of diabetes mellitus has been placed in patients previously treated with dietary treatments, who had received oral antidiabetic agents or insulin or had a fasting plasma glucose value higher than 126 mg/dL.

All subjects signed informed consent before study. The study was conducted in accordance with the guidelines of the Declaration of Helsinki for human research and the guidelines of our local ethics committee.

### 4.2. Assay of RAGE Genotype

Genomic DNA was extracted from ethylenediaminetetraacetic acid (EDTA)-treated blood using the Maxwell 16 Blood DNA purification kit (Promega, Madison, WI, USA). The –374T/A polymorphism of the RAGE promoter was analyzed by polymerase chain reaction (PCR) restriction fragment length polymorphisms (RFLPs). In brief, a section of the RAGE promoter that contains the –374T/A variant was amplified by PCR. The sequences were 5′-CCTGGGTTTAGTTGAGATTTTTT-3′ for upstream and 5′-GAAGGCACTTCCTCGGGTTCT-3′ for downstream primers. The conditions of amplification were: 94 °C for 2 min; 30 cycles at 94 °C for 30 s, 58 °C for 30 s, 72 °C for 1 min; and finally 10 min at 72 °C. The PCR product (671 bp) was then subjected to Tsp509 I (New England Biolabs, Beverly, MA, USA) digestion for 4 h at 65 °C, whereas the PCR product was resolved by electrophoresis on 3% agarose gels. Genotypes were scored according to the patterns of DNA bands. Treatment of the T allele with Tsp509 I gave rise to five fragments of 284 bp, 217 bp, 110 bp, 44 bp, and 16 bp. In the case of the A allele, digestion resulted in four fragments of 327 bp, 284 bp, 44 bp, and 16 bp. To reduce the possibility of genotyping errors, all results were scored by two independent investigators blinded to clinical status of subjects. All ambiguous samples were analyzed a second time.

### 4.3. Statistical Analysis

The hypothesis of normal distribution for continuous variables was tested according to Kolmogorov-Smirnov statistics. Continuous variables are expressed as mean ± standard deviation in the case of Gaussian distribution and as median in the case of variables with non-normal distribution. The data were analyzed using the Student t test for paired. All categorical variables are reported as absolute (*n*) and relative (%) frequencies. The associations between the categorical variables were evaluated using the chi-square test. Statistical significance was defined as *p* < 0.05.

## 5. Conclusions

Our research showed a higher prevalence of the AA genotype in male population of subjects aged ≥90 years who have reached that age in good state of health. These results confirm the hypothesis that longevity is the result of a good functioning of the immune system and a presumable hyper-expression of variants of anti-inflammatory genes of immunity. The differences in the genetic regulation of inflammatory processes may influence the presence of age-related disorders. Surprisingly, the prevalence of the AA genotype is lower in the long-living female population than in the male one, in which this genotype would appear to have a more protective role.

Thus, AA genotype would not seem to have a role in determining a better aging in the female gender. In the general population, women are more long-living than men, so one wonders if the endocrine system cannot have a protective role even in those subjects without this genotype. Further research is needed to identify other agents involved in healthy aging.

## Figures and Tables

**Table 1:** Demographic, clinical and biochemical characteristics of subject included in the study.

	Subjects with ≥90 years (*n* = 119)	Subjects with <80 years (*n* = 135)	*p* Value
Age, years	93 ± 3	62 ± 10	<0.0001
Male, *n* (%)	28 (24%)	56 (41%)	NS
Hypertension, *n* (%)	36 (30%)	41 (30%)	NS
Smoking, *n* (%)	15 (4%)	26 (19%)	<0.01
Total Cholesterol (mg/dL)	183 ± 33	147 ± 59	0.0029
Triglycerides (mg/dL)	97 ± 18	138 ± 11	NS
HDL Cholesterol (mg/dL)	44 ± 14	50 ± 21	NS
Glucose (g/dL)	104 ± 30	90 ± 21	0.01

**Table 2:** –374T/A genotype and allele frequencies in middle-aged and long-living subjects.

RAGE –374T/A	Middle-aged individuals (*n* = 135)	Subjects with ≥90 years (*n* = 119)	*p* Value
**Genotype**			

AA	29 (21%)	29 (24%)	NS
AT	67 (50%)	54 (45%)	NS
TT	39 (36%)	36 (30%)	NS

**Allele**			

A	125 (46%)	112 (47%)	NS
T	145 (54%)	126 (53%)	

**Table 3:** Allelic and genotypic distribution of –374 T/A polymorphisms in middle-aged males and long-living ones.

RAGE –374T/A	Middle-aged males (*n* = 56)	Males with ≥90 years (*n* = 28)	*p* Value
**Genotype**			

AA	10 (18%)	12 (43%)	0.028
AT	28 (50%)	6 (21%)	0.023
TT	18 (32%)	10 (36%)	NS

**Allele**			

A	48 (43%)	30 (54%)	NS
T	64 (57%)	26 (46%)	
